# Editorial: Dietary Factors, Epigenetics and Their Implications for Human Obesity

**DOI:** 10.3389/fendo.2020.00601

**Published:** 2020-08-28

**Authors:** Marie-Claude Vohl, María M. Malagón, Bruno Ramos-Molina

**Affiliations:** ^1^Centre Nutrition, Santé et Société (NUTRISS)-Institut sur la Nutrition et les Aliments Fonctionnels (INAF), Université Laval, Quebec, QC, Canada; ^2^Department of Cell Biology, Physiology, and Immunology, Maimonides Biomedical Research Institute of Cordoba, Reina Sofia University Hospital, Cordoba, Spain; ^3^CIBER Physiopathology of Obesity and Nutrition (CIBERobn), Institute of Health “Carlos III”, Madrid, Spain; ^4^Biomedical Research Institute of Murcia (IMIB-Arrixaca), Murcia, Spain

**Keywords:** epigenetics, obesity, diet, nutrients, DNA methylation

Obesity has reached a pandemic scale worldwide, mainly caused by changes in lifestyles that include the regular consumption of energy-dense foods and a reduction of physical activity. It is currently considered a serious threat to human health because of its close association with multiple comorbidities such as type 2 diabetes, hypertension, dyslipidemia, atherothrombotic cardiovascular disease, and cancer. There is now a growing body of evidence suggesting that epigenetic mechanisms may underlie the development of obesity-associated metabolic disorders (1–3). Among factors involved in epigenetic modifications, the role of nutrients on DNA methylation and accessibility have gathered increasing attention because methyl group donors and related molecules contributing to DNA methylation are derived from food. In particular, specific dietary behaviors lead to changes in epigenetic patterns and regulate gene expression via epigenetic modifications thereby affecting obesity-related metabolic disorders (4, 5). Remarkably, obesity has been associated with epigenetic alterations in tissues involved in the regulation of energy balance and glucose homeostasis such as adipose tissue, endocrine pancreas, liver, and skeletal muscle, suggesting that epigenetics could have a direct impact on the pathogenesis of obesity and its comorbidities (1, 2, 6).

The present Research Topic includes four original articles, which provide relevant new evidence about the epigenetic aspects of obesity and related comorbidities and their regulation through nutritional cues. Edillor et al. aimed to investigate the hepatic DNA methylome changes in response to a high-fat and high-sucrose (HFHS) diet challenge in a panel of genetically diverse mouse strains to understand whether these changes are reversible when mice were fed a low-fat diet to induce weight loss. The authors found that weight loss occurs more rapidly than DNA methylation changes, and that differences in weight loss in response to a HFHS diet challenge may be associated to DNA methylation changes. Accordingly, these authors suggest that obesity-induced changes to DNA methylation may be involved in unresolved metabolic adaptation or epigenetic “memory” following body weight loss, which may have potential implication for predicting the success of body weight loss, and subsequent risk of future weight regain.

Another interesting concept covered in this Research Topic is related to the relationship between epigenetic processes and aging. In this regard, Arpón et al. studied the role of obesity phenotypes, insulin resistance or dyslipidemia on DNA methylation and epigenetic age acceleration, finding that the relationship between markers of the metabolic syndrome and accelerated epigenetic age was sex dependent. Thus, whereas in men epigenetic age acceleration was associated to an accumulation of visceral fat and the presence of insulin resistance, in women the mechanisms were more related to dyslipidemia and inflammation markers. The authors concluded that the identified associations between accelerated epigenetic age and disease markers may contribute to a better understanding of the development of age-related diseases, and that sex is a key factor to take into account to implement precision strategies and to manage healthy aging.

Aberrant epigenetic modifications have long been reported to play a major role in tumorigenesis. ZNF577 is a member of the zinc finger protein family, which has been found to be frequently hypermethylated and silenced in different tumor types, including obesity-related breast cancer. Within this Research Topic Lorenzo et al. reported that the methylation pattern of *ZNF577* in circulating leukocytes was highly related to adiposity and menopausal status in women with confirmed breast cancer. Remarkably, *ZNF577* methylation levels correlated with paired leukocytes and breast tumor biopsies. Another interesting finding was that adherence to the Mediterranean diet, most specifically to fish consumption, appeared to modulate the methylation levels of *ZNF577* in blood leukocytes independently of the effects of age and BMI. The authors concluded that the ZNF577 methylation blood leukocytes level may be used as a biomarker of environmental factors such as adiposity, age, and diet on breast cancer, and a suitable therapeutic target in precision nutrition and medicine.

Emerging evidence have established a link between maternal gestational diabetes, which is defined by glucose intolerance not present or recognized prior to pregnancy, and epigenetic alterations in the newborn. Gestational diabetes is also characterized by alterations in the materno-fetal transfer of essential nutrients. In this regard, Sánchez-Campillo et al. demonstrated that the protein Major Facilitator Superfamily Domain containing 2A (MFSD2a), a primary carrier for the uptake of long chain polyunsaturated fatty acids (especially docosahexaenoic acid, DHA) in several organs such as the brain and that is highly expressed in the placenta, was present in human blood, and that pregnant women with gestational diabetes displayed lower blood and placenta levels of MFSD2a as well as reduced cord/maternal serum ratio of DHA, suggesting a compromised DHA materno-fetal transport. This reduction in MFSD2a in blood correlated with the postnatal infant head circumference during the first 6 months of life, implying that disturbed MFSD2a levels during pregnancy might affect the development of the fetus and the neonatal brain.

Overall, articles in this Research Topic have covered new insights on the link between nutrition, epigenetics, and human obesity and related metabolic disorders, which could help to establish new therapeutic approaches based on epigenetic strategies.

## Author Contributions

All authors listed have made a substantial, direct and intellectual contribution to the work, and approved it for publication.

## Conflict of Interest

The authors declare that the research was conducted in the absence of any commercial or financial relationships that could be construed as a potential conflict of interest.
